# A park-based group mobility program for older adults with difficulty walking outdoors: a quantitative process evaluation of the Getting Older Adults Outdoors (GO-OUT) randomized controlled trial

**DOI:** 10.1186/s12877-023-04524-7

**Published:** 2023-12-11

**Authors:** Ruth Barclay, Sandra C. Webber, Francine Hahn, C. Allyson Jones, Nancy E. Mayo, Shajicaa Sivakumaran, Yixiu Liu, Philip D. Chilibeck, Nancy M. Salbach

**Affiliations:** 1 Department of Physical Therapy, University of Manitoba, Winnipeg, MB Canada; 2 Department of Physical Therapy, University of Alberta, Edmonton, AB Canada; 3 School of Physical and Occupational Therapy, McGill University, Montreal, QC Canada; 4 Department of Physical Therapy, University of Toronto, 160-500 University Avenue, Toronto, ON M5G 1V7 Canada; 5 Department of Community Health Sciences, Max Rady College of Medicine, University of Manitoba, Winnipeg, MB Canada; 6 College of Kinesiology, University of Saskatchewan, Saskatoon, SK Canada; 7 Rehabilitation Sciences Institute, University of Toronto, Toronto, ON Canada; 8 The KITE Research Institute, University Health Network, Toronto, ON Canada

**Keywords:** Older adults, Outdoor walking, Physical activity, Randomized controlled trial, Task-oriented training, Parks, Community exercise program, Process evaluation

## Abstract

**Background:**

Process evaluations of randomized controlled trials (RCTs) of community exercise programs are important to help explain the results of a trial and provide evidence of the feasibility for community implementation. The objectives of this process evaluation for a multi-centre RCT of outdoor walking interventions for older adults with difficulty walking outdoors, were to determine: 1) implementation fidelity (the extent to which elements of the intervention were delivered as specified in the original protocol) and 2) participant engagement (the receipt of intervention components by the participants) in the Getting Older Adults Outdoors (GO-OUT) trial.

**Methods:**

GO-OUT participants attended an active 1-day workshop designed to foster safe, outdoor walking skills. After the workshop, 190 people at 4 sites were randomized to an outdoor walk group (OWG) (*n* = 98) which met 2x/week for 10 weeks, or the weekly reminders (WR) group (*n* = 92) which received a phone reminder 1x/week for 10 weeks. The OWG had 5 components – warm-up, continuous distance walk, task-oriented walking activities, 2^nd^ continuous distance walk, and cool-down. Data on implementation fidelity and participant engagement were gathered during the study through site communications, use of standardized forms, reflective notes of the OWG leaders, and accelerometry and GPS assessment of participants during 2 weeks of the OWG.

**Results:**

All sites implemented the workshop according to the protocol. Participants were engaged in all 8 activity stations of the workshop. WR were provided to 96% of the participants in the WR intervention group. The 5 components of the OWG sessions were implemented in over 95% of the sessions, as outlined in the protocol. Average attendance in the OWG was not high – 15% of participants did not attend any sessions and 64% of participants in the OWG attended > 50% of the sessions. Evaluations with accelerometry and GPS during week 3 and 9 OWG sessions suggest that participants who attended were engaged and active during the OWG.

**Conclusions:**

This process evaluation helps explain the main study findings and demonstrates the flexibility required in the protocol for safe and feasible community implementation. Future research could explore the use of additional behaviour change strategies to optimize attendance for community implementation.

**Trial registration:**

ClinicalTrials.gov NCT03292510 Date of registration: September 25, 2017.

**Supplementary Information:**

The online version contains supplementary material available at 10.1186/s12877-023-04524-7.

## Background

For older adults, walking is a popular form of physical activity [1, 2]. While all walking is beneficial for health, outdoor walking in particular has specific advantages. Research suggests people who are active outdoors have greater levels of physical activity compared to individuals who are physically active indoors only [3, 4], and exercising in natural environments is associated with positive feelings, increased energy and decreased depression [5]. In older adults, spending at least 30 min outdoors each day lowers depressive symptoms, reduces the fear of falling, and improves levels of self-reported functioning [6, 7]. Low frequency of going outdoors is associated with increased risks of experiencing musculoskeletal pain [7], problems with sleep [7], decline in physical function [7, 8], and social isolation [9]. Unfortunately, data from the Canadian Longitudinal Study on Aging indicates that 33% of individuals 65 years of age and older walk outside fewer than 3 days per week [10].

Patla and Shumway-Cook proposed a widely accepted theoretical framework of eight dimensions of individual and environmental factors important for older adults’ ambulation in the community [11]. Research supports that individual characteristics (e.g., limitations in lower extremity strength, balance and general fitness [12, 13]; fear of and/or low self-efficacy for community mobility [12, 14]) and environmental considerations (e.g., lack of social support [15], transportation issues [15], poor neighborhood walkability [12, 13], and extreme weather [11, 13, 16]) limit levels of outdoor walking among older adults. However, only recently have researchers begun to investigate outdoor walking interventions designed to address one or more of the dimensions proposed in the framework. A recent systematic review [17] found only five studies targeting community dwelling older adults practicing walking in outdoor settings that included at least one mobility task as outlined by Patla and Shumway-Cook [11].

The Getting Older Adults Outdoors (GO-OUT) study was a two-group randomized controlled trial (RCT; registered 25/09/2017 with ClinicalTrials.gov NCT03292510) designed to build physical capacity and self-efficacy to walk outdoors in older adults with self-reported limitations in outdoor walking for any reason, including physical impairments and low motivation [18]. Participants entered the study in two cohorts, one in the spring of 2018 and the second in spring 2019. All participants attended a 1-day workshop to build knowledge and skills important for safe walking outside. They were then randomized to either the task-oriented, multi-component outdoor walk group (OWG, *n* = 98), which met twice a week for 10 weeks, or the weekly reminders (WR, *n* = 92) group, which received a phone reminder once a week for 10 weeks. Outcomes were measured at baseline, 3 months, 5.5 months and 12 months. Participants in cohort 2 only completed self-report measures at 12 months due to the COVID-19 pandemic [19]. The primary outcome was minutes spent walking outdoors (measured from combined accelerometry and global positioning system (GPS) data). Secondary outcomes were derived from scores on multiple measures related to the constructs of walking capacity, health-promoting behaviour, and successful aging.

Results of the GO-OUT RCT showed no statistically significant differences between the OWG and WR group in change from baseline in minutes spent walking outdoors [20]. The OWG showed improvements in walking capacity from 0–3 months superior to the WR group; all other comparisons were not significant [20]. The driver of improvement in walking capacity was an improvement in walking self-efficacy [20].

Assessing implementation fidelity of the delivery of intervention components and participant engagement by receipt of intervention are important to establish internal validity in RCTs, provide context to study results, and provide insight into how interventions can be improved [21–23]. In this way, process evaluation may help explain results, determine mechanisms of change and explain how different contexts affect implementation and outcomes [23]. It is especially important to perform process evaluation in RCTs conducted at multiple sites because the same intervention may be delivered and received in diverse ways [24]. Implementation fidelity and participant engagement are often evaluated through observing and/or interviewing participants and research assistants, reviewing videotaped sessions, and by analyzing project documentation (e.g., participant attendance records, notes explaining protocol deviations) [25]. In trials that involve physical activity or walking in particular, activity duration and distance are typically estimated by program leaders [26], however, it is possible [27] and recommended [28, 29], to use devices such as pedometers or accelerometers to more accurately and comprehensively assess whether a walking intervention was delivered as it was intended. Qualitative methodology is also regularly used in the evaluation of complex interventions [30]. A qualitative process evaluation of GO-OUT was conducted to explore participants’ experiences with the interventions and examine contextual factors that may have affected outcomes [31]. Two themes were identified: “Holding Me Accountable to Walk More Frequently” which represented experiences of participants in both the OWG and WR group, and “We Walked Farther, With More Ease and Confidence, and We Felt Better” which described experiences only of participants in the OWG [31].

The objectives of this quantitative process evaluation were to determine: 1) implementation fidelity (the extent to which elements of the intervention were delivered as specified in the original protocol) and 2) participant engagement (the receipt of intervention components by the participants). The results of the process evaluation will help to better understand the main study results, and provide information on the feasibility of implementing the outdoor walking program.

## Methods

### Design

The quantitative process evaluation was a planned component of the GO-OUT study and is described in the protocol [18]. The focus of the process evaluation was on intervention fidelity and participant engagement.

### Interventions

The GO-OUT study was a 2-group RCT conducted in Edmonton, Winnipeg, Toronto and Montreal, Canada. Ethics boards from the four academic institutions associated with the research sites approved the study and participants signed a consent form.

All participants were asked to attend an interactive workshop in groups of up to 18 participants prior to randomization [18, 32]. Over the course of 5 h they rotated in small groups (2–3 individuals) through eight stations: Canadian physical activity guidelines for older adults; monitoring exercise intensity and safety; setting SMART (i.e., specific, measurable, achievable, realistic and timely) goals; pedometer use; Nordic pole walking; foot care, footwear, proper walking patterns; falls prevention; and postural awareness and balance exercises [18]. All participants received a pedometer and a workbook to keep. The workbook included detailed information relevant to the workshop stations. Graduate and undergraduate students in health professional programs or health professionals experienced with working with older adults and people with chronic conditions provided the education and led discussion at each station.

Participants in the WR group received a phone call (or e-mail if participants could not be reached) from the study coordinator each week for 10 weeks after attending the workshop. These scripted reminders were designed to reinforce information that was covered in the workshop, encourage participants to work towards their goals for outdoor walking, and answer questions.

The OWG program consisted of twice-weekly walking sessions held in neighborhood parks for 10 weeks. Sessions for the OWGs ran during summer months from June to August, except in site 4, where three groups ran August to early October. The OWG program was based on theory; activities were designed to build competence in established dimensions of community mobility including distance, temporal factors, terrain, physical load, attentional demands, postural transitions, and traffic density [11, 33]. Sessions were supervised by walk group leaders who were health professionals (e.g., physical therapist, kinesiologist), assisted by other trained staff, to achieve a 3:1 ratio of participants to facilitators. Each session lasted for one hour and included 5 components: a 10-min warm-up, a continuous distance walk, task-oriented walking activities (e.g., walking and changing direction, stepping to the side, starting and stopping, walking on slopes/over curbs), a second continuous distance walk, and a 10-min cool down. The walking activities differed between weeks and addressed two or more dimensions of outdoor ambulation [11]. Target distances for the two continuous distance walks in each session were the same for both walks, but they differed depending on whether the participant’s baseline comfortable 10 m walk test (10mWT) gait speed was < 0.8 m/s or ≥ 0.8 m/s [34, 35]. For example, the target distance for the continuous walks in week 3 was 225 m for participants with a baseline comfortable 10mWT gait speed < 0.8 m/s and 425 m for participants with 10mWT gait speeds ≥ 0.8 m/s. In week 9, the target distances were 400 m and 600 m, respectively.

### Participants

Community-dwelling older adults were eligible for the GO-OUT study if they were: ≥ 65 years of age, living independently in the community, able to walk at least one block (~ 50 m) without a break, with or without a walking aid and without supervision, self-reported difficulty walking outdoors, limited time (< 75 min/week) spent walking outdoors during good weather months from May to October (note: this criterion was dropped for the second cohort as participants had difficulty with estimating the amount of time they walked outdoors), willingness to sign a waiver or obtain physician clearance to exercise, mentally competent (scored ≥ 18/22 on Mini-Mental State Exam telephone version), and able to understand and speak English [18]. Older adults were excluded if they: self-reported engaging in physical activity for ≥ 150 min per week by estimating time spent in their usual physical activities, were currently receiving rehabilitation to improve their walking, were at high risk for falls according to the American Geriatric Society criteria [18, 36]. We also asked individuals if they were available for the workshop date and for at least 5 of the 10 weeks of the OWG program.

### Staff training and materials

Staff training was carried out for the evaluators, workshop facilitators, OWG leaders and assistants, and weekly reminder callers. Evaluators at all sites completed evaluator training by videoconferencing for 2 h, led by the Toronto site. The evaluator manual, which included standard operating procedures, was reviewed in detail. Each site ran a practice session. The workshop facilitator training at each site consisted of a 2-h in-person review and discussion of the participant workshop booklet and the workshop facilitator guide, as well as practice of the content of each station. The Winnipeg site provided OWG training to leaders at all sites. The 90-min training was provided by teleconference and videoconferencing, recorded, and made available to leaders and assistants to review at a later time. Review of the OWG facilitator guide included theoretical background, safety considerations and week by week details of the OWG intervention. Videos were created and available for staff which highlighted safety and use of Nordic Poles. Training for the WR callers included review of the WR facilitator guide which detailed the WR script and discussion topics for each week.

### Data collection: implementation fidelity

Evaluating the delivery of the intervention involved monitoring completion of training of workshop facilitators and OWG leaders and the extent to which each intervention component (i.e., workshop, OWG, WR) was delivered as planned at each site. For the OWG, sessions could be cancelled for safety reasons due to weather: temperature of 30 degrees Celsius or greater; winds of greater than 30 km per hour; poor air quality (based on high-risk classification by the Government of Canada) [37]; or rain (based on the judgment of the OWG leader).

Table 1 outlines the process evaluation plan. Information about the variables used in the process evaluation was obtained through site communication, use of standardized forms, and reflective notes of the OWG leaders. Originally, the telephone WR was conceived of as a measure of participant engagement with the study [18], however, if participants were not available to talk on the phone when the weekly call was made and they had an e-mail address, then the reminder content was e-mailed to them. In this way, provision of the 10 weekly telephone or e-mail reminders was tracked as a measure of implementation fidelity.

**Table 1:** Process evaluation plan

Process Evaluation	Variable	Indicator	Source
Implementation fidelity	Workshop training	Implementation of workshop facilitator training session (yes/no)	Site communication
	Workshop implementation	Implementation of stations 1–8 (yes/no)	Site completed standardized form
	OWG leader training	Attendance of OWG leaders at training session	Communication from site coordinators or leaders
	OWG implementation	Implementation of 2 walk sessions per week for 10 weeks	OWG leaders completed standardized forms
		Implementation of 5 components per session (warm-up, walk one, walking activity, walk two, and cool-down) twice each week as described in OWG Facilitator Guide	OWG leaders completed standardized forms and reflective notes
		Level of outdoor walking activity achieved during OWG	Accelerometer and GPS data collected during walking sessions in weeks 3 and 9
		Use of Nordic poles during session 5 as per protocol	OWG leaders completed standardized forms
	WR implementation	Implementation of 10 weekly telephone or e-mail reminders	Sites completed standardized form
Participant engagement	Engagement with workshop	Participant attendance	Sites completed standardized form
		Participant completion of workshop stations 1–8	Sites completed standardized form
	Engagement with OWGs	Participant attendance	OWG leaders completed standardized forms
		Accompanied by a non-participant each session	OWG leaders completed standardized forms

*Abbreviations*: *OWG* Outdoor walk group, *WR* Weekly reminders, *GPS* Global positioning system

Device-based information related to the walk sessions was collected with ActiGraph activity monitors (GT3X + , ActiGraph LLC, Pensacola, FL) and Global Positioning System (GPS) devices (QStarz BT-Q1000XT A-GPS Travel Recorders) that were used in two of the OWG sessions (one session in week 3 and one session in week 9) to measure the distance, duration, and speed of the two continuous distance walks completed in that session. Participants wore both devices on an elastic belt which was positioned over the right hip. ActiLife 6 software (version 6.13.3; ActiGraph LLC) was used to initialize activity monitors to collect data at a sampling rate of 100 Hz [38]. The GPS devices logged GPS data every second.

### Data collection: participant engagement

Participant engagement included evaluating participants’ levels of participation with the workshop and with the outdoor walking sessions, outlined in Table 1. Site reports indicated the number of participants who attended workshops and the number of stations completed at each workshop. Standardized forms completed by OWG leaders after each walk session were reviewed to obtain information about levels of attendance, completion of the 5 components, as well as whether or not participants were accompanied by a non-participant.

### Data analysis

Data from site communications and standardized forms were summarized and presented as frequencies and percentages. Sociodemographic data were presented as mean and standard deviation (SD), or frequency and percentages.

To obtain device-based information about the walking that occurred (during one session in week 3 and one session in week 9), GT3X + activity monitor data were downloaded using ActiLife’s low frequency extension (LFE) filter. Using the LFE filter is recommended to improve the monitor’s detection of steps in individuals who walk slowly [39, 40]. The timing and duration of each continuous distance walk were determined from the GT3X + data. The process used to manually identify the continuous distance walks in the accelerometer data has been previously described [41]. In brief, we determined that the cadence threshold of ≥ 40 steps/minute demonstrated high sensitivity, specificity and positive predictive value to detect the walking bouts in the week 3 and week 9 walking sessions [41]. A walking bout was defined as a period of activity ≥ 5 min in duration with a cadence ≥ 40 steps/minute for each minute, allowing for a maximum of 1 min below threshold. Mean gait speeds during outdoor continuous distance walks were calculated by dividing the distance travelled (measured by GPS) by the walking bout duration (determined by accelerometer data). A custom R program (https://www.r-project.org) was developed to identify start and end times in 60 s epoch files. GPS data were downloaded and start/end times were marked as identified in the GT3X + data. We used the Haversine formula to compute the distance covered in each walk based on latitude and longitude data [42]. Mean walking speed for each continuous distance walk was calculated as distance (metres) divided by time (seconds).

For week 3 and 9 data, the median and 25^th^/75^th^ percentile values were presented for distance, duration and speed variables. Walking speed data (indoor and outdoor evaluations) were normally distributed so a two-tailed paired t-test was used to determine differences between gait speeds recorded indoors in the 10mWT and mean gait speed recorded during the outdoor continuous distance walks in weeks 3 and 9. Bland Altman plots [43] were used to assess agreement between protocol specifications and distances walked during continuous walks. Distances achieved by participants were plotted on the x-axes and difference between these distances and protocol-specified target distances were plotted on the y-axes.

## Results

### Participants

Participants’ sociodemographic and health characteristics are presented by intervention group and site in Table 2. Over the two years of recruitment (spring 2018 and spring 2019), 190 participants were randomized, 98 to the OWG (75.5% women, mean age 75.3 (SD 6.9) years), and 92 to the WR group (70.7% women, mean age 74.2 (SD 7.4) years). The seven most commonly reported health conditions are reported in Table 2. Other health conditions experienced by the participants less frequently included diabetes, myocardial infarction and other cardiac conditions, stroke, glaucoma, orthopedic issues, osteoporosis, Parkinson’s disease, and fibromyalgia.

**Table 2:** Sociodemographic and health characteristics of participants by intervention group and site (*n* = 190)^a^

Characteristic n (%)	Outdoor Walk Group						Weekly Reminders Group					
	**n**	**Site 1** **(** ***n*** ** = 26)** Y1 = 11 Y2 = 15	**Site 2** **(** ***n*** ** = 28)** Y1 = 7 Y2 = 21	**Site 3** **(** ***n*** ** = 26)** Y1 = 9 Y2 = 17	**Site 4** **(** ***n*** ** = 18)** Y1 = 6 Y2 = 12	**Pooled** **(** ***n*** ** = 98)** Y1 = 33 Y2 = 65	**n**	**Site 1** **(** ***n*** ** = 25)** Y1 = 9 Y2 = 16	**Site 2** **(** ***n*** ** = 25)** Y1 = 8 Y2 = 17	**Site 3** **(** ***n*** ** = 24)** Y1 = 11 Y2 = 13	**Site 4** **(** ***n*** ** = 18)** Y1 = 4 Y2 = 14	**Pooled** **(** ***n*** ** = 92)** Y1 = 32 Y2 = 60
Participant type												
Individual	98	20 (76.9)	24 (85.7)	22 (84.6)	12 (66.7)	78 (79.6)	92	19 (76.0)	23 (92.0)	22 (91.6)	12 (66.7)	76 (82.6)
Dyad		6 (23.1)	4 (14.3)	4 (15.4)	6 (33.3)	20 (20.4)		6 (24.0)	2 (7.7)	2 (8.3)	6 (33.3)	16 (17.2)
Age in years												
Mean (SD)	98	74.7 (6.9)	73.5 (6.3)	77.6 (7.1)	76.7 (6.6)	75.3 (6.9)	92	70.9 (4.0)	73.7 (6.4)	80.3 (8.3)	73.7 (7.1)	74.2 (7.4)
Range		65–94	65–86	65–91	67–90	65–94		64–79	66–86	66–93	63–84	63–93
Sex												
Female	98	18 (69.2)	20 (71.4)	23 (88.5)	13 (72.2)	74 (75.5)	92	17 (68.0)	17 (68.0)	18 (75.0)	13 (72.2)	65 (70.7)
Male		8 (30.8)	8 (28.6)	3 (11.5)	5 (27.8)	24 (24.5)		8 (32.0)	8 (32.0)	6 (25.0)	5 (27.8)	27 (29.3)
BMI												
Mean (SD)	98	29.6 (6.4)	31.8 (6.8)	26.8 (5.1)	29.3 (6.3)	29.6 (6.4)	90	28.6 (3.9)	29.9 (7.4)	27.8 (5.3)	32.1 (7.7)	29.3 (6.2)
Range		18.6–49.3	18.8–49.3	19.3–36.4	20.1–40.8	18.6–49.3		18.6–35.4	15.6–44.7	20.4–45.3	21.8–49.4	15.6–49.4
Frailty classification^b^												
Not frail	95	10 (38.5)	13 (46.4)	4 (16.0)	3 (18.8)	30 (31.6)	87	13 (52.0)	10 (43.5)	6 (26.1)	4 (25.0)	33 (38.0)
Pre-frail		16 (61.5)	11 (39.3)	17 (68.0)	12 (75.0)	56 (59.0)		12 (48.0)	12 (52.2)	14 (60.9)	12 (75.0)	50 (57.8)
Frail		0 (0)	4 (14.3)	4 (16.0)	1 (6.3)	9 (9.5)		0 (0)	1 (4.4)	3 (13.0)	0 (0)	4 (4.6)
Walking aid used daily												
None	98	20 (76.9)	22 (78.6)	13 (50.0)	15 (83.3)	70 (71.4)	92	22 (88.0)	21 (84.0)	14 (58.3)	15 (83.3)	72 (78.3)
Single point cane		4 (15.4)	4 (14.3)	6 (23.1)	2 (11.1)	16 (16.3)		2 (8.0)	2 (8.0)	5 (20.8)	2 (11.1)	11 (12.0)
Walking poles		1 (3.8)	1 (3.6)	1 (3.8)	1 (5.6)	4 (4.1)		1 (4.0)	1 (4.0)	1 (4.2)	0 (0)	3 (3.3)
4-wheeled walker		1 (3.8)	1 (3.6)	6 (23.1)	0 (0)	8 (8.2)		0 (0)	1 (4.0)	4 (16.7)	1 (5.6)	6 (6.5)
Health conditions												
None	98	3 (11.5)	1 (3.6)	1 (3.8)	1 (5.6)	6 (6.1)	92	2 (8.0)	2 (8.0)	1 (4.2)	0 (0)	5 (5.4)
Arthritis		15 (57.7)	23 (82.1)	22 (84.6)	10 (55.6)	70 (71.4)		13 (52.0)	17 (68.0)	17 (70.8)	11 (61.1)	58 (63.0)
Hypertension		9 (34.6)	15 (53.6)	9 (34.6)	8 (44.4)	41 (41.8)		12 (48.0)	12 (48.0)	12 (50.0)	9 (50.0)	45 (48.9)
Impaired hearing		8 (30.8)	7 (25.0)	11 (42.3)	6 (33.3)	32 (32.7)		2 (8.0)	6 (24.0)	9 (37.5)	2 (11.1)	19 (20.7)
Cataracts		4 (15.4)	11 (39.3)	9 (34.6)	5 (27.8)	29 (29.6)		4 (16.0)	10 (40.0)	9 (37.5)	5 (27.8)	28 (30.4)
Thyroid problem		5 (19.2)	7 (25.0)	7 (26.9)	6 (33.3)	25 (25.5)		6 (24.0)	6 (24.0)	6 (25.0)	0 (0)	18 (19.6)
Respiratory conditions^c^		8 (30.8)	5 (17.9)	6 (23.1)	5 (27.8)	24 (24.5)		3 (12.0)	6 (24.0)	2 (8.3)	3 (16.7)	14 (15.2)
Cancer		5 (19.2)	5 (17.9)	9 (34.6)	4 (22.2)	23 (23.5)		6 (24.0)	2 (8.0)	7 (29.2)	0 (0)	15 (16.3)

Abbreviations: Y = cohort (year); SD = standard deviation

^a^All values are n (%) unless otherwise stated

^b^Frailty classification determined by Cardiovascular Health Study Frailty Index [47]

^c^ Respiratory conditions included emphysema, chronic obstructive pulmonary disease, bronchitis, asthma, pulmonary fibrosis

Each site offered between 2 and 4 OWG over the period of the study. The size of each OWG varied by site, ranging from 6–15 participants, with a 3:1 participant to facilitator ratio. This represents a protocol deviation, as the recommended size of an individual group was nine participants and one site had two walk groups that were larger than that. Table 3 presents characteristics of participants in each OWG by site. At site 1, OWG sessions were held at two parks and at site 3, three parks were used over the 10 weeks, whereas at sites 3 and 4, all sessions were held in the same location. The OWG leaders were experienced healthcare professionals (e.g., physical therapist, kinesiologist) familiar working with people with mobility limitations.

**Table 3:** Outdoor walk group participant characteristics by session at each site

Variable	Site 1		Site 2				Site 3			Site 4		
	Year 1 Group 1	Year 2 Group 1	Year 1 Group 1	Year 2 Group 1	Year 2 Group 2	Year 2 Group 3	Year 1 Group 1	Year 2 Group 1	Year 2 Group 2	Year 1 Group 1	Year 2 Group 1	Year 2 Group 2
n	11	15	7	6	9	6	9	8	9	6	6	6
Participant Characteristics												
Age Mean (SD)	76.6 (7.2)	72.3 (7.1)	76.0 (6.4)	72.8 (7.3)	69.8 (3.4)	74.8 (7.6)	80.4 (6.7)	72.3 (4.1)	78.1 (7.6)	79.7 (7.9)	76.5 (6.1)	72.0 (4.3)
Range	65–86	65–94	68–85	66–85	65–74	69–86	71–91	65–77	67–88	72–90	67–82	67–79
Sex n (%)												
Female	8 (72.7)	10 (66.7)	5 (71.4)	5 (83.3)	5 (55.6)	5 (83.3)	8 (88.9)	8 (100)	7 (77.8)	6 (100)	4 (66.7)	3 (50.0)
Male	3 (27.3)	5 (33.3)	2 (28.6)	1 (16.7)	4 (44.4)	1 (16.7)	1 (11.1)	0	2 (22.2)	0	2 (33.3)	3 (50.0)
Walking aid, used daily												
No	7 (63.6)	13 (86.7)	4 (57.1)	5 (83.3)	7 (77.8)	6 (100)	3 (33.3)	7 (87.5)	3 (33.3)	4 (66.7)	6 (100)	5 (83.3)
Yes	4 (36.4)	2 (13.3)	3 (42.9)	1 (16.7)	2 (22.2)	0 (0)	6 (66.6)	1 (12.5)	6 (66.6)	2 (33.3)	0 (0)	1 (16.7)

*Abbreviations*: *n*. Number, *Year* cohort, *Group* walk group number within each site, *SD* Standard deviation

### Implementation fidelity

#### Workshops

Fifteen workshops were completed and facilitators across sites completed workshop training. Sites delivered all eight stations at each workshop.

#### Outdoor walk groups (OWGs)

Twelve OWGs were delivered in total. Sites engaged a total of 7 OWG leaders and 17 OWG assistants throughout the study. All leaders and assistants received and were asked to review the detailed week by week facilitator guide; each leader was required to use the document for reference throughout the study. Six of the seven (86%) OWG leaders attended the training session.

Two OWGs conducted all 20 (100%) planned sessions, 8 groups ran 17–19 (85–95%) sessions, 1 group conducted 16 (80%) sessions and 1 group ran 14 (70%) sessions. Reasons for cancelled sessions included rain (79.2%), poor air quality (12.5%), and high winds (8.3%).

The percentage of OWG sessions in which each of the five planned activity components was delivered was: warm-up 98.9%, first continuous distance walk 98.5%, walking skills 97.6%, second continuous distance walk 95.2%, cool down 97.1%.

Across all sites, we collected activity monitor/GPS data on 62 participants in week 3 and 59 participants in week 9. All people who attended one of the days in week 3 and 9 were evaluated. There were many more participants with baseline gait speeds ≥ 0.8 m/s (*n* = 54 with week 3 data, *n* = 52 with week 9 data) compared to individuals with gait speeds < 0.8 m/s (*n* = 8 with week 3 data, *n* = 7 with week 9 data). Data from distances achieved by all participants in the week 3 and week 9 continuous walks are illustrated in Bland Altman plots in Supplementary files Figures S1-S4. In most instances, distances walked in the continuous distance walks were within 100 m of the target distance. However, in week 3, the mean difference between distances walked and target distances was > 100 m for site 3 participants where the majority of participants did not attain target distances (mean distance in walk 1 = 263.5 m, mean distance in walk 2 = 299.5 m). For week 9, (walk 1) mean distances achieved in site 1 were more than 400 m greater than target. In site 1 reflective notes, the walking leader noted that the two continuous distance walks were combined together, a protocol deviation which resulted in one longer walk, reflected in distances achieved (Figure S3). In site 3, mean distances were more than 200 m below target for the first continuous distance walk in week 9 and more than 400 m below target for the second continuous distance walk.

We compared mean outdoor walking speed achieved at week 3 and week 9 with baseline comfortable gait speed derived from the 10mWT test completed indoors. Results are presented in Table 4. Participants with 10mWT speeds ≥ 0.8 m/s walked significantly slower outdoors compared to indoors (*p* < 0.001).

**Table 4:** Walking speeds indoors and outdoors^a^

Outdoor walk group week and level of walking ability	n	Indoor 10mWT baseline gait speed (comfortable) (m/s)	Outdoor gait speed (m/s)	*P *value (Paired t-test)
Week 3, < 0.8 m/s	8	0.69 (0.08)	0.61 (0.10)	0.05
Week 9, < 0.8 m/s	7	0.70 (0.09)	0.67 (0.18)	0.65
Week 3, ≥ 0.8 m/s	51	1.10 (0.20)	0.67 (0.15)	< 0.001
Week 9, ≥ 0.8 m/s	43	1.12 (0.21)	0.75 (0.21)	< 0.001

Mean (SD)

^a^This table includes participants who attended a week 3 session with two continuous distance walks and participants who attended a week 9 session with two continuous distance walks

Table 5 presents median values of walking distance, speed and duration for the two continuous distance walks in week 3 and week 9 for 43 participants with complete data at both time points. This table does not include data related to the protocol deviation at site 1 described above.

**Table 5:** Distance, speed and duration of continuous distance walks in week 3 and 9 of outdoor walk group

Parameter	Site	n	Week 3	Week 9
			Median (P_25_, P_75_)	Median (P_25_, P_75_)
**Walk 1**				
Distance (m)	1	4	424.30 (398.70, 436.03)	490.95 (482.75, 504.43)
	2	16	434.00 (402.55, 454.20)	494.77 (476.68, 508.68)
	3	11	244.60 (229.00, 337.60)	329.91 (306.02, 358.63)
	4	12	416.40 (397.58, 669.28)	458.92 (380.74, 622.07)
	pooled	43	403.40 (341.30, 436.15)	478.02 (352.25, 511.46)
Speed (m/s)	1	4	0.74 (0.72, 0.77)	0.91 (0.88, 0.92)
	2	16	0.70 (0.65, 0.74)	0.75 (0.68, 0.83)
	3	11	0.46 (0.44, 0.61)	0.47 (0.45, 0.58)
	4	12	0.57 (0.55, 0.99)	0.83 (0.78, 0.94)
	pooled	43	0.65 (0.55, 0.75)	0.75 (0.60, 0.87)
Duration (min)	1	4	9.10 (8.43, 9.83)	9.05 (8.78, 9.55)
	2	16	10.00 (9.23, 12.00)	10.50 (8.00, 13.00)
	3	11	9.00 (9.00, 9.90)	11.00 (11.00, 12.00)
	4	12	12.00 (11.00, 12.33)	10.00 (7.78, 11.25)
	pooled	43	10.00 (9.00, 12.00)	11.00 (8.35, 12.00)
**Walk 2**				
Distance (m)	1	4	440.10 (434.73, 454.60)	578.90 (573.08, 583.80)
	2	16	432.40 (400.85, 460.55)	538.58 (481.81, 558.15)
	3	11	292.90 (254.05, 345.60)	161.98 (83.33, 199.71)
	4	12	363.15 (305.73, 444.85)	430.31 (416.17, 587.63)
	pooled	43	391.40 (305.65, 445.90)	431.28 (306.33, 568.89)
Speed (m/s)	1	4	0.67 (0.65, 0.70)	0.83 (0.81, 0.85)
	2	16	0.61 (0.58, 0.65)	0.78 (0.64, 0.95)
	3	11	0.54 (0.47, 0.74)	0.45 (0.25, 0.49)
	4	12	0.56 (0.55, 0.76)	0.94 (0.75, 0.98)
	pooled	43	0.61 (0.54, 0.72)	0.77 (0.53, 0.92)
Duration (min)	1	4	11.05 (10.70, 11.43)	11.65 (11.25, 12.08)
	2	16	11.95 (11.00, 12.48)	11.00 (9.00, 12.75)
	3	11	9.00 (9.00, 9.00)	6.00 (5.00, 7.00)
	4	12	10.95 (9.30, 11.00)	9.60 (7.83, 12.00)
	pooled	43	10.90 (9.10, 11.30)	9.20 (7.10, 11.65)

Table includes 43 participants who had accelerometry and GPS data for walk 1 and walk 2 at both week 3 and 9

*m* Metres, *m/s*  Metres per second, min Minutes, *P*_*25*_ 25^th^ percentile, *P*_*75*_ 75^th^ percentile

During the week 5 session which included a long continuous distance walk with Nordic Poles, the overall mean pooled percentage of participants who attended the OWGs and used Nordic poles was 73.9%. Individual session use of Nordic poles in week 5 varied from 0–100%. Participants who did not use the poles had a variety of reasons. Some participants did not like using Nordic Poles and in some cases, the walk group leaders may have decided that walking poles were not safe for some participants who used walking aids, such as a walker.

#### Weekly reminders group (WR)

Ten weekly reminders were delivered to 96% of participants in site 1, 80% in site 2 and 79% in site 3, and 28% in site 4. Two percent of participants received zero weekly reminders. See Table 6 for the number of WR delivered at each site and pooled across sites. Overall, participants received an average of 88.4% of reminders by phone, and 11.6% by email. At site 4, the caller documented that calls were attempted five times each week to participants. If the participant did not answer, either an email was not attempted by the caller or the participant did not have an email address.

**Table 6:** Number of weekly reminders delivered

Total weekly reminders provided out of 10	Site 1 (*n* = 25)	Site 2 (*n* = 25)	Site 3 (*n* = 24)	Site 4 (*n* = 18)	Pooled (*n* = 92)
	**n (%)**				
10	24 (96.0)	20 (80.0)	19 (79.2)	5 (27.8)	68 (73.9)
9	1 (4)	1 (4.0)	3 (12.5)	5 (27.8)	10 (10.9)
8	0 (0)	0 (0)	1 (4.2)	2 (11.1)	3 (3.3)
7	0 (0)	1 (4.0)	0 (0)	0 (0)	1 (1.1)
6	0 (0)	0 (0)	0 (0)	1 (5.6)	1 (1.1)
5	0 (0)	1 (4.0)	0 (0)	3 (16.7)	4 (4.3)
4	0 (0)	1 (4.0)	0 (0)	1 (5.6)	2 (2.2)
3	0 (0)	0 (0)	0 (0)	0 (0)	0 (0)
2	0 (0)	0 (0)	0 (0)	1 (5.6)	1 (1.1)
1	0 (0)	0 (0)	0 (0)	0 (0)	0 (0)
0	0 (0)	1 (4.0)	1 (4.2)	0 (0)	2 (2.2)

### Participant engagement

#### Workshops

The percentage of participants who attended the workshop was 90.8% in the OWG and 96.7% in the WR group. On average, each of the eight stations in the workshop were completed by 86–89% of participants in the OWG and 87%-89% in the WR group.

#### Outdoor walk groups (OWGs)

Mean attendance at the 20 OWG sessions was 43.8% in site 1, 71.2% in site 2, 58.4% in site 3, and 84.9% in site 4. Table 7 presents the number of OWG sessions attended. For the 98 participants assigned to OWGs, 15.3% attended 0 sessions, 20.4% attended 1–10 sessions, 34.7% attended 11–15 sessions, and 29.6% attended 16–20 sessions. Reasons for non-attendance included illness, vacation, other commitments, soreness, transportation difficulties, and employment. The mean number of participants in OWGs who attended sessions with a non-participant was 3.8%, with the range being 0.3% to 10.8% across sites.

**Table 7:** Number of outdoor walk group sessions attended

Total sessions attended out of 20	Site 1 (*n* = 26)	Site 2 (*n* = 28)	Site 3 (*n* = 26)	Site 4 (*n* = 18)	Pooled (*n* = 98)
	**n (%)**				
16–20	3 (11.5)	14 (50.0)	3 (11.5)	9 (50.0)	29 (29.6)
11–15	7 (26.9)	9 (32.1)	12 (46.2)	6 (33.0)	34 (34.7)
6–10	7 (26.9)	1 (3.6)	4 (15.4)	2 (0.11)	14 (14.3)
1–5	2 (7.7)	0 (0)	4 (15.4)	0 (0)	6 (6.1)
0	7 (26.9)	4 (14.3)	3 (11.5)	1 (5.6)	15 (15.3)

## Discussion

Findings from this quantitative process evaluation of the GO-OUT trial demonstrated that the workshop was successfully implemented at multiple sites as per protocol and participants were engaged in the workshop. The provision of a high percentage of the WR to participants provides evidence of implementation fidelity of the WR intervention. The five components of the OWG sessions, as outlined in the protocol, were successfully implemented with participants in the study. However, average attendance was not high. Evaluations with accelerometry and GPS during week 3 and 9 OWG sessions suggest that participants who attended were engaged and active during the OWG. This process evaluation demonstrates feasibility for community implementation and can help to explain the main study findings.

### Explaining main study findings

The primary finding in the main trial [20] of no difference between groups in minutes walked outdoors may be explained in part by attendance. Participant attendance and the number of sessions cancelled due to weather could have potentially decreased the intervention effect. The intention to treat analysis of the primary trial included all randomized participants. Fifteen percent of the participants in the OWG did not attend any sessions and only 64% of the participants attended > 50% of the sessions. In contrast, 85% of the WR group participants received 90–100% of the 10 reminders. It is important to note that the WR group entailed passive involvement, whereas the OWG required more active involvement. In a study of a supervised OWG using Nordic poles on trails with flat surfaces and hills, and a control group of indoor circuit training, attendance was higher in the control group [44]. In the GO-OUT study, the receipt of weekly reminders in the WR control group was also higher than attendance at the OWG; in both studies the experimental group occurred in park settings. At study entry, participants confirmed they would be generally available for at least 5 weeks of the 10-week OWG intervention, but at this point sites had not yet finalized the specific times and days of the OWG sessions. Thus, some people may have missed sessions due to scheduling conflicts; indeed, one of the reasons given for absence was “other commitments”. Attendance at the OWG program implemented in community settings may be higher as typically program schedules are verified before programs are advertised.

The secondary GO-OUT finding of a greater increase in OWG walking capacity from 0–3 months (driven by walking self-efficacy) [20], is supported by the indication that participants were active during the OWG sessions. Average distance walked outdoors during the OWG sessions at week 3 to week 9 showed an increased distance, as per the protocol, and over 95% of all the walk groups completed all five planned activities (warm-up, continuous distance walk, task-oriented walking activities, 2^nd^ continuous distance walk, and cool-down). Participants in the OWG had the opportunity to practice walking in a variety of ways by completing two continuous distance walks and a task-oriented walking activity each session and demonstrated increased walking distance and walking speed from week 3 to week 9, which may have led to the observed increase in walking capacity, and specifically self-efficacy. Participants from the OWG who were part of the qualitative process evaluation confirmed this with comments that they were able to walk farther, for a longer time, and felt more confident, and “felt better” after being a part of the OWG [31].

Participants who walked indoors at speeds of ≥ 0.8 m/s walked significantly slower outdoors compared to their observed comfortable gait speed evaluated indoors at baseline. The 10mWT was conducted indoors on a level surface, with no breaks in walking. While walking outdoors, it is possible, that participants took short breaks (< 1 min in duration) during the continuous distance walks, slowed their pace to deal with uneven terrain or match their speed with another walker. All outdoor walking speeds noted in Tables 4 and 5 fall within the range of ‘slow pace’, as identified in a systematic review of outdoor gait speed [45].

### Feasibility for community implementation

The OWG facilitator guide outlined a weekly increase in distance walked and the inclusion of five activities each session; both aspects were implemented and appear feasible for community implementation. A community based OWG will provide social support to participants, which is an important aspect of walking outdoors for many older adults [46]. Overall, it was noted that the distances walked in the continuous distance walks of the OWG were within 100 m of the target distance, suggesting feasibility of the weekly OWG guidelines for community implementation. It was apparent from OWG leader notes and evaluation of week 3 and 9 distances, that deviations to the facilitator guide protocol occurred on occasion. These deviations highlight the need for flexibility of the protocol to enhance safety by adapting to individual participants based on their abilities and preferences. For example, one site combined both continuous distance walks into one walk based on the preferences of the participants and leaders, while at another site (site 3), participants walked under the distance suggested in week 9 by on average 200 m and 400 m in walk 1 and 2 respectively. This represented needed safety adaptations, as site 3 had the highest proportion of participants identified as frail (16%) and 4 wheeled-walker users (23%) compared to the other sites. The target walking distances were determined prior to the start of the trial, based on whether a participant’s 10mWT gait speed was < 0.8 m/s or ≥ 0.8 m/s [18]. Walk leaders’ reflective notes documented instances of participants with slower baseline gait speeds wanting to challenge themselves by attempting the longer walking distance, and/or wanting to walk with another person who was walking the longer distance. Sometimes participants with faster baseline gait speeds did not attempt the longer target distance (e.g., experiencing arthritic pain, and/or if the weather was warm or windy). This reasonable flexibility within the protocol will be beneficial for safe and responsible implementation of the outdoor walking program into a community setting.

On average, 74% of participants who attended the week 5 session used the Nordic Poles for the long continuous distance walk. We had noted that participants had a variety of reasons for not using Nordic Poles and the walk group leaders may have decided against the use of walking poles for some participants due to safety concerns. This finding supports the importance of flexibility for implementation in a community setting focused on both preference and safety of the participants.

### Strengths and limitations

Strengths of this quantitative process evaluation included the use of multiple measures to evaluate OWG participant engagement and implementation fidelity. Measures included both facilitator-reported measures and device-based measures of the walking achieved during two weeks of the intervention. A limitation of this process evaluation is that findings represent the unique circumstances of the participants (those with self-perceived walking difficulties), and may not be relevant in other contexts. Reasons for non-attendance of the workshop, OWG or non-receipt of the WR were not consistently recorded; future projects could revise procedures to make this aspect clearer. This study consisted of four sites with participants of different abilities, and site leaders made choices for comfort and safety of the participants, leading to some variations in the interventions by site.

### Clinical and research implications

Training guides and detailed procedures were developed for all study staff. The ability of the OWG leader to modify as appropriate for safety concerns was also built in. These components may make the interventions sustainable for future community implementation. However, it would be important to meet with community groups to review and revise the documents for language clarity and level of expertise to be useful for community implementation. Incorporating additional behaviour change and motivational strategies into the intervention could help with community implementation and attendance in OWG interventions. Aspects of our procedures which could be applied to future process evaluations were: identifying the evaluation process a priori at the time of the protocol publication and incorporating the aspects of self-report and device-based assessment in the process evaluation.

## Conclusions

The planned GO-OUT quantitative process evaluation identified aspects of implementation fidelity and participant engagement in the trial. The workshop, OWG, and WR were implemented as per the study protocol, however attendance in the OWG was not high. The study findings help to explain the results of the main GO-OUT study and provide evidence for the potential implementation of the OWG program in a community setting.

## Consent for publication

Not applicable.

### Supplementary Information

**Additional file 1: Figures S1-S4. **Bland Altman plots for walking distance week 3 and week 9. **Figure S1. **Bland Altman plots for walking distance Week 3 Walk 1. **Figure S2. **Bland Altman plots for walking distance Week 3 Walk 2. **Figure S3. **Bland Altman plots for walking distance Week 9 Walk 1. **Figure S4. **Bland Altman plots for walking distance Week 9 Walk 2. 

## Data Availability

The datasets generated and/or analysed during the current study are not publicly available as the participant consent forms did not address open public access to the data and due to limitations of the research ethics approval by the health sciences research ethics boards at the University of Toronto, University of Manitoba, University of Alberta and McGill University. Data are available from the corresponding author on reasonable request and subject to research ethics boards review.

## Abbreviations

GO-OUT: Getting Older Adults Outdoors
GPS: Global positioning system
OWG: Outdoor walk group
WR: Weekly reminders
COVID-19: Coronavirus 2019
RCT: Randomized controlled trial
SMART: Specific, measurable, achievable, realistic and timely
10mWT: 10 metre walk test
m: Metre
LFE: Low frequency extension
SD: Standard deviation
IQR: Inter-quartile range
