# Supplementary material for: A park-based group mobility program for older adults with difficulty walking outdoors: a quantitative process evaluation of the Getting Older Adults Outdoors (GO-OUT) randomized controlled trial
Source: BMC Geriatr. 2023 Dec 11;23:833. doi: 10.1186/s12877-023-04524-7 (PMC10712059; doi:10.1186/s12877-023-04524-7)

**Supplementary Files**

**Figures S1-S4- Bland Altman plots for walking distance week 3 and week 9**

How to interpret figures: Each color represents a different walking group that met and walked together over the 10 week sessions. For example, Site1 plots for week 3 include 2 colors because they ran two walking groups, whereas Site 2 data include 4 colors to designate their 4 walking groups. On plots for all walks (walk 1 and walk 2 in week 3, and walk 1 and 2 in week 9), the data points are largely clustered by color (i.e., walking group attended). Vertical alignment of triangle and circle shapes (representing participants with different baseline walking speeds) with the same colour (e.g., black triangle and black circle Site 1, week 3 walk 1) indicate that participants walked the same distance, perhaps together, despite having different goals for the session. Distance goals were based on baseline 10mWT gait speeds.

The solid horizontal line on each graph represents the mean difference between the actual distances walked and the target distances. The dotted horizontal line on each graph represents zero difference

**Figure S1 - Bland Altman plots for walking distance Week 3 Walk 1**

n=60 (missing = 2)


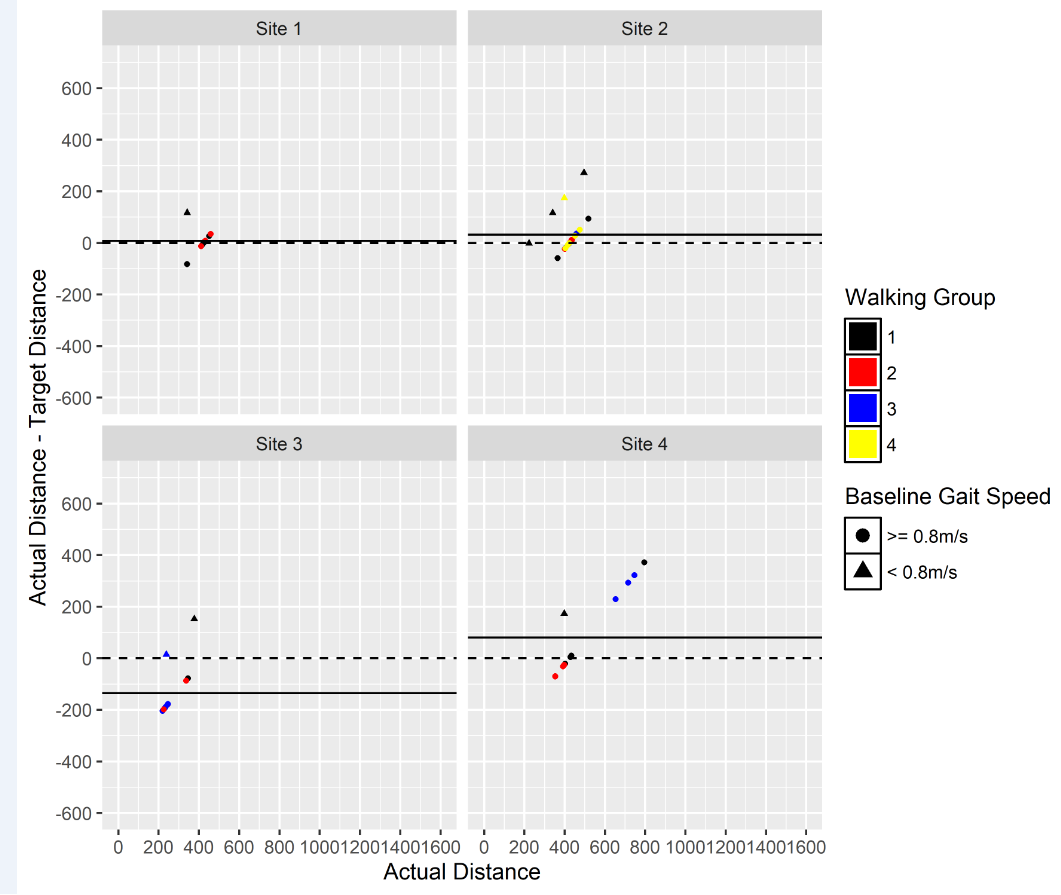


**Figure S2 - Bland Altman plots for walking distance Week 3 Walk 2**

n=61 (missing = 1)


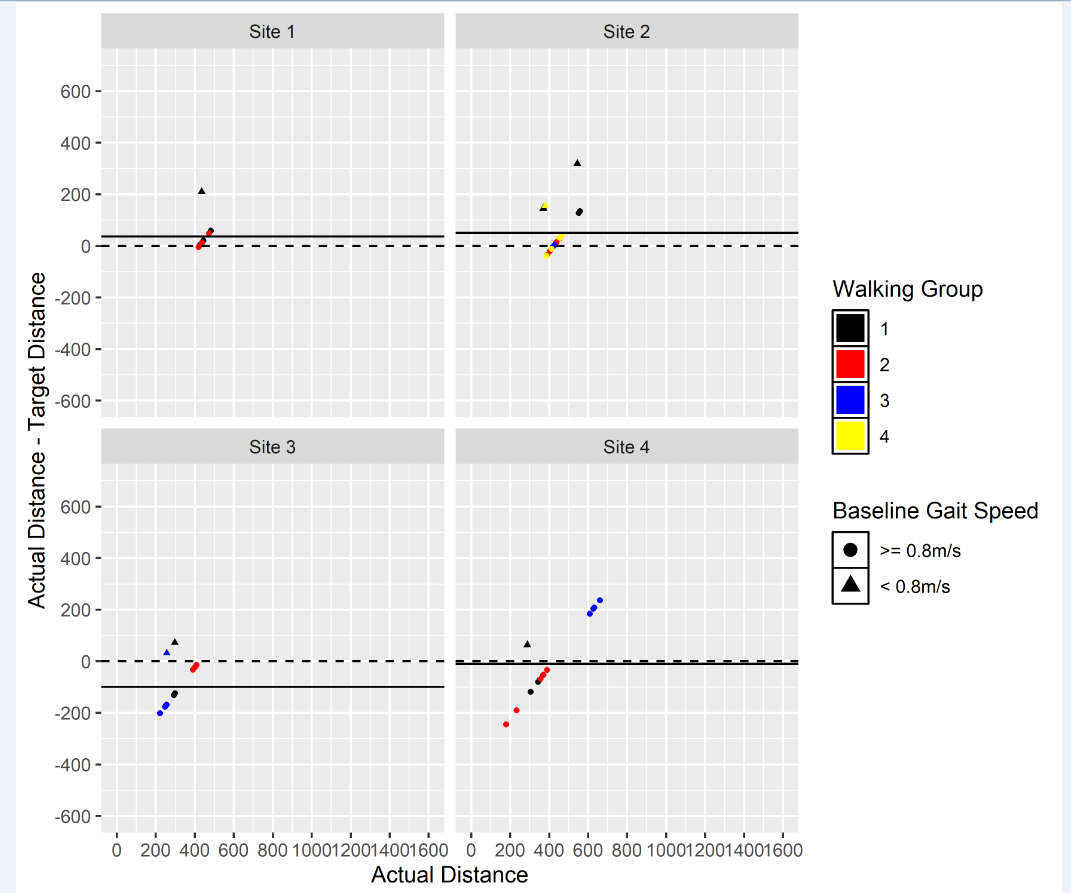


**Figure S3 - Bland Altman plots for walking distance Week 9 Walk 1**

n=59


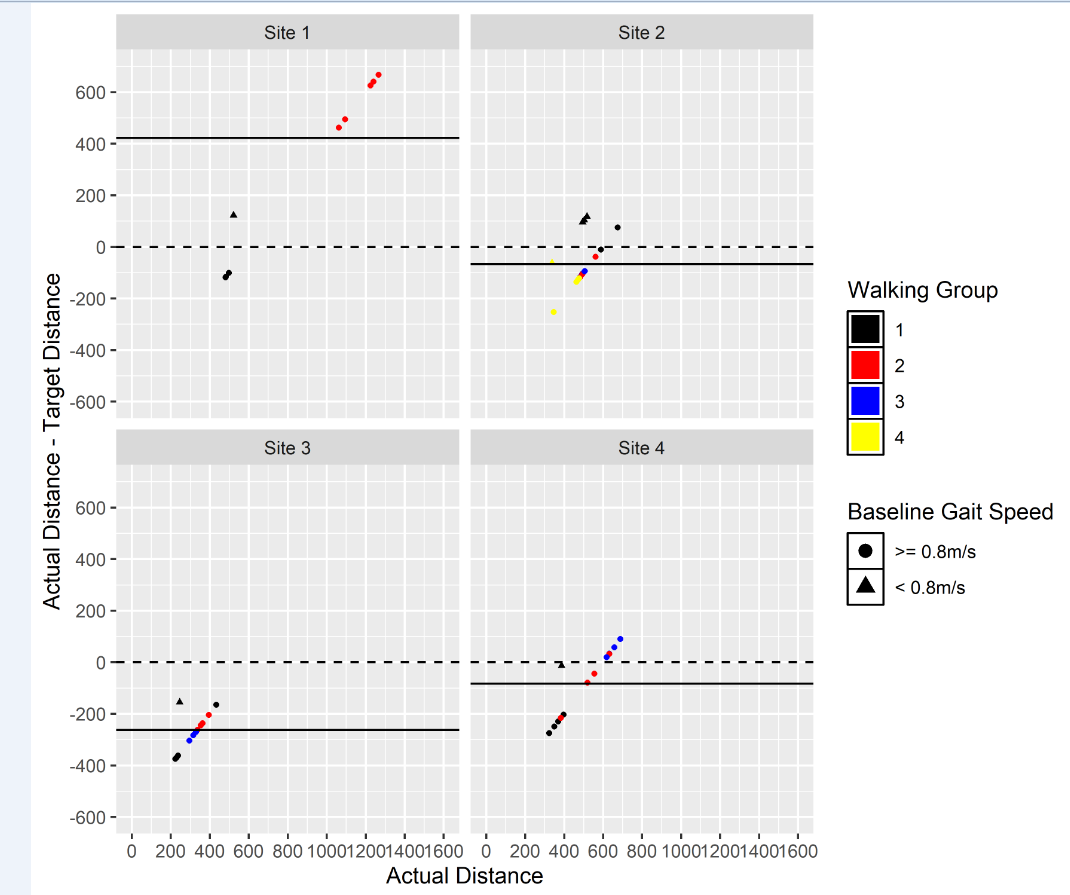


Note for Site 1 **-** two red dots representing 955.18m and 950.30, respectively were omitted from the graph.

**Figure S4 - Bland Altman plots for walking distance Week 9 Walk 2**

n=51 (missing = 8)


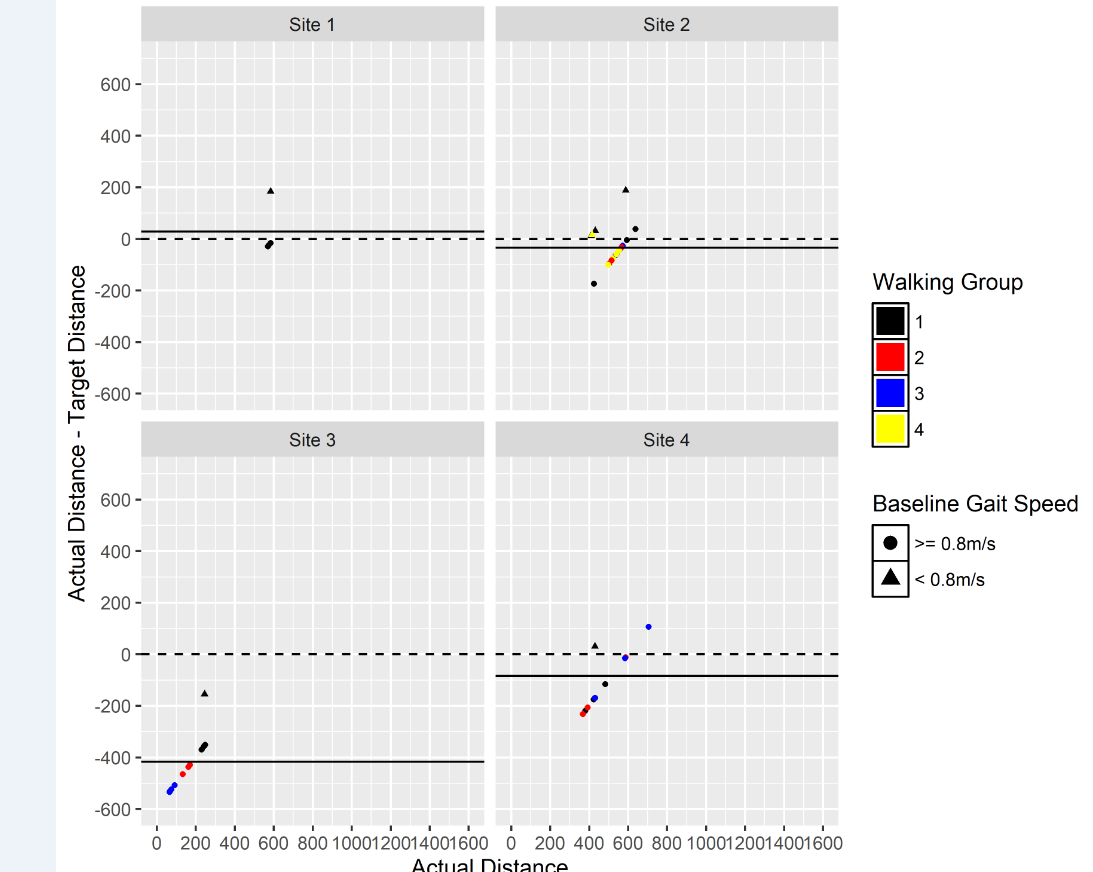

Supplement: Supplementary file 1 — Additional file 1: Figures S1-S4. Bland Altman plots for walking distance week 3 and week 9. Figure S1. Bland Altman plots for walking distance Week 3 Walk 1. Figure S2. Bland Altman plots for walking distance Week 3 Walk 2. Figure S3. Bland Altman plots for walking distance Week 9 Walk 1. Figure S4. Bland Altman plots for walking distance Week 9 Walk 2. [file 12877_2023_4524_MOESM1_ESM.docx]
